# Supplementary material for: Content-rich biological network constructed by mining PubMed abstracts
Source: BMC Bioinformatics. 2004 Oct 8;5:147. doi: 10.1186/1471-2105-5-147 (PMC528731; doi:10.1186/1471-2105-5-147)
Supplement: Additional File 5 — The original Chilibot query results of the term "long-term potentiation (LTP)" and 22 other terms, limiting the latest references analyzed to the years 1990, 1995, 2000, and 2004. [file 1471-2105-5-147-S5.bz2 › chilibotAdditionalFile5/ltp2000/html/ACTIN_SYNAPTOPHYSIN.html]

 


 **ACTIN** and **SYNAPTOPHYSIN** 
  
Found 77 abstracts in PubMed,  **30 abstracts were retrieved and analyzed**.  


---

 Search Google  |
 PDF files only 
|  EDU domain only 

---

**Interactive relationship** (e.g. stimulation, inhibition, etc)

- As with neurites in differentiated neuroblastoma cells, processes induced by the PKCepsilon PSC1V3 protein contained alpha tubulin, neurofilament 160, and F  **actin** , but the PKCepsilon PSC1V3 induced processes lacked the synaptic markers  **synaptophysin**  and neuropeptide Y.  Ref: 10330401 J Cell Biol, 1999
- After training, pp60c src from hippocampus showed enhanced interactions with synaptic proteins such as synapsin I,  **synaptophysin** , and the type 2 N methyl d aspartate receptor, as well as the cytoskeletal protein  **actin** .  Ref: 10884433 Proc Natl Acad Sci U S A, 2000
- Both tumors stained for vimentin, neuron specific enolase, and  **synaptophysin** , but were negative for CD99, muscle specific  **actin** , and myogenin.  Ref: 10496592 Mod Pathol, 1999
- Tumor cells stained positively for smooth muscle  **actin**  and vimentin, but were negative for cytokeratin, S 100, desmin,  **synaptophysin** , factor VIII related antigen, and neuron specific enolase.  Ref: 10643990 Vet Pathol, 2000

**Parallel relationship** (e.g. studied together, co-existance, homology, etc.)

- Immunohistochemical studies showed focal myoglobin reactivity in one case, while cytokeratin, vimentin, S100 protein, chromogranin,  **synaptophysin** , neurofilaments, leu enkephalin, desmin, smooth muscle  **actin**  and muscle specific  **actin**  were negative in both cases.  Ref: 9416489 Histopathology, 1997
- Immunohistochemical staining was positive for vimentin, epithelial membrane antigen, chromogranin A,  **synaptophysin** , neuron specific enolase, S 100 protein, and muscle  **actin** .  Ref: 10632935 Pathol Int, 1999
- METHODS Histology, immunocytochemistry for neurofilaments, neurone specific enolase,  **synaptophysin** , glial fibrillar acidic protein, S100 protein, and smooth muscle alpha  **actin** , and electron microscopic examinations on the resected colon.  Ref: 10517919 Gut, 1999
- Most of the stromal spindle cells were positive for smooth muscle  **actin** , neuron specific enolase and  **synaptophysin** .  Ref: 10808558 Ultrastruct PatholUltrastruct Pathol, 1999
- To clarify these issues, the immunoprofiles of 59 SPTs were defined by applying a battery of antibodies against cytokeratin, vimentin, neuron specific enolase,  **synaptophysin** , chromogranin A, tyrosine hydroxylase, AAT, LeuM1, Ki M1P, smooth muscle  **actin** , CD34, alpha inhibin, calretinin, placental alkaline phosphatase, and progesterone and estrogen receptors.  Ref: 10881741 Virchows Arch, 2000
- In the present study, the secreted isoform of N CAM SEC N CAM,  **synaptophysin** , and  **actin**  proteins were measured in the hippocampus of controls, suicide victims, and patients with bipolar disorder or schizophrenia by quantitative Western immunoblotting.  Ref: 10523820 Mol Psychiatry, 1999
- Three cases 75% were positive for  **synaptophysin** , and reactivity for alpha smooth muscle  **actin** , epithelial membrane antigen, neuron specific enolase and CD99 O 13 was present in two cases 50% each.  Ref: 11012987 Pathol Int, 2000
- The battery of antibodies included LCA, CD3, OPD 4, CD34, UCHL 1, L 26, KP 1, MAC 387, Factor XIIIa, Leu 7, S 100 protein, HMB 45, GFAP,  **synaptophysin** , neurofilament protein, ubiquitin, vimentin, type IV collagen, laminin, fibronectin, Factor VIII related antigen, Desmin M, smooth muscle  **actin** , cytokeratin 7, cytokeratin 20, AEI AE3, CAM 5.2, EMA, GCDFP, Ki 67, and PCNA.  Ref: 9747658 J Dermatol Sci, 1998
- Double staining for alpha smooth muscle  **actin**  and  **synaptophysin** , detected by confocal laser scanning microscopy, unequivocally demonstrated colocalization of both markers in lobular stellate cells.  Ref: 10595912 Am J Pathol, 1999
- The neoplasms showed striking polyphenotypic immunoreactivity, including that for vimentin, glial fibrillary acidic protein, epithelial membrane antigen, cytokeratins,  **synaptophysin** , chromogranin, and smooth muscle  **actin** .  Ref: 9737241 Am J Surg Pathol, 1998
- The  **synaptophysin**   **actin**  ratio was significantly decreased in schizophrenia P = 0.014 as compared to controls.  Ref: 10523820 Mol Psychiatry, 1999
- Immunohistochemical stains for CD3, Leu 7, S 100 protein, type IV collagen, laminin, Factor VIII related antigen, GFAP,  **synaptophysin** , neurofilament protein, ubiquitin, smooth muscle  **actin** , vimentin, Desmin M, cytokeratin 7, cytokeratin 20, AE1 AE3, CAM 5.2, Ki 67 and PCNA showed consistent cross reactivity.  Ref: 9747658 J Dermatol Sci, 1998
- Each of the cases showed the following immunophenotype S 100,  **synaptophysin** , vimentin, alpha smooth muscle  **actin** , CD 30, HMB 45, and pankeratin.  Ref: 11100813 J Cutan Pathol, 2000
- The immunohistochemical profile included negative stains for S 100, CD34, factor XIIIa, CD68, HMB45, cytokeratins, and EMA, strongly positive stains for neuron specific enolase NSE,  **synaptophysin** , and chromogranin A, and focally positive ones for NKI C3 and alpha smooth muscle  **actin** .  Ref: 10405481 J Dermatol, 1999
- Highly purified antibodies were used to reveal the distribution of intermediate filaments vimentin, desmin, alpha smooth muscle  **actin** , histiocytic antigens alpha 1 antitrypsin, lysozyme, neural and neural crest associated antigens neuron specific enolase, chromogranin A, S 100,  **synaptophysin** , glial fibrillary acidic protein, neurofilaments in the adult pulp tissue.  Ref: 9578642 Minerva StomatolMinerva Stomatol, 1999
- Among them, forty six cases were immunostained using antibodies against vimentin, CD34, muscle specific  **actin**  MSA, smooth muscle  **actin**  SMA, neuron specific enolase NSE, S 100 protein,  **synaptophysin**  SYN, cytokeratin CK, CEA, LCA.  Ref: 10920995 Zhonghua Zhong Liu Za Zhi, 1998
- Monoclonal antibodies reactive with cytokeratin AE1 AE 4betaE12, Cam 5.2 as well as a variety of other cellular antigens vimentin,  **actin** , desmin, chromogranin,  **synaptophysin** , CD57, neuron specific enolase NSE, and S100 were used in an immunoperoxidase method with paraffin embedded tissue to phenotypically characterize 23 cases of BSCC, 10 cases of SCUC, and 15 cases of ACC.  Ref: 9635682 Hum Pathol, 1998
- Thirty seven of 39 cases reacted for cytokeratin using a cocktail of 3 monoclonal antibodies, 39 39 for desmin, 24 25 for epithelial membrane antigen, 22 27 for vimentin, 18 25 for neuron specific enolase, 10 15 for CD5 19 for  **synaptophysin** , 1 22 for chromogranin, 3 19 for muscle specific  **actin** , 3 16 for alpha smooth muscle  **actin** , 11 16 for CD1 12 for CA 12 17 for CD9 10 for MOC 3 6 for NB8 7 for Ber EP4, and 8 9 for the Wilms tumor protein.  Ref: 9808124 Am J Surg Pathol, 1998
- Immunohistochemical staining showed positive immunoreactivity for neuronespecific enolase, vimentin, p53 30%, p21 10% and cyclin D1 20%, and negative immunoreactivity for CD45, cytokeratins, S 100 protein, glial fibrillary acidic protein,  **synaptophysin** , chromogranin, calcitonin, serotonin, thyreoglobulin, desmin, myosin,  **actin** , HMB 45, pRB, p16 and BCL 2.  Ref: 10208257 Graefes Arch Clin Exp Ophthalmol, 1999
